# Social support and epigenetic aging at the intersections of race, ethnicity, and gender: findings from NHANES 1999–2002

**DOI:** 10.1016/j.ssmph.2025.101892

**Published:** 2025-12-04

**Authors:** Hanyang Shen, Nicole Gladish, Andres Cardenas, Belinda L. Needham, David H. Rehkopf

**Affiliations:** a Department of Epidemiology and Population Health, Stanford University, Palo Alto, CA, USA; b Department of Pediatrics, Stanford University, Palo Alto, CA, USA; c Department of Epidemiology, University of Michigan, Ann Arbor, MI, USA; d Department of Health Policy, Stanford University, Palo Alto, CA, USA; e Department of Medicine (Primary Care and Population Health), Stanford University, Palo Alto, CA, USA; f Department of Sociology, Stanford University, Palo Alto, CA, USA

## Abstract

• The connection of social support and epigenetic aging varies markedly by person.
• Sufficient emotional support is linked to slower epigenetic aging among Black men.
• Having close friends was associated with slower epigenetic aging among Black women.
• Epigenetic aging was slower among married Black and Mexican American men.
• Hannum and Weidner clocks were the most sensitive to social support variability.

## 1 Introduction

Social support has been suggested to have a consequential influence on both short- and long-term health outcomes. Experimental studies suggest that social support is associated with improved depressive and anxiety symptoms (Hou et al., 2022), less risky and better disease management behaviors (Shushtari et al., 2023; Spencer-Bonilla et al., 2017; Swinkels et al., 2023), increased health-related information and knowledge, and stress reduction (Hogan et al., 2002). Observational studies have also reported associations between a lack of social support and increased risk of long-term incidence of cardiovascular disease (Freak-Poli et al., 2021), stroke (Freak-Poli et al., 2021), diabetes (Van Dam et al., 2005), cancer (Nausheen et al., 2009), cognitive decline (Salinas et al., 2021), and mortality (Shor et al., 2013; Wang et al., 2023). Among these studies, the most consistent result is the association with increased mortality risk. A 2010 meta-analysis of 148 studies involving over 300,000 participants found that perceived social support and being married were associated with a 35 % and 33 % reduced likelihood of mortality, respectively (Holt-Lunstad et al., 2010). A 10-year follow-up study of 206 Finnish participants also found that women in the lowest tertile of perceived emotional support had a 2.5 times higher risk of death compared to those in the highest tertile (Lyyra & Heikkinen, 2006).

### 1.1 Social support theory

Social support is broadly defined by Cohen and Syme as the resources provided by others (Cohen, 1985). Social support, being viewed as resources, can be described as both a structural support and a functional support (Cohen & Wills, 1985). Structural support quantifies the existence of the support by measurable scales, and functional support refers to qualitative information of the support, including access and availability to social support resources. For example, the size and frequency of contact highlight the structural characteristics of social support, evaluating social integration and beyond of a person's social relationship. There are potentials for social support to have positive or negative effects on health, and social support is hypothesized to positively affect health both directly and indirectly (Taylor & others, 2011; Thoits, 1985). Directly, it promotes companionship, intimacy, acceptance, and encouragement which can promote confidence, security, sense of belonging, sense of control, and life purpose of a person. These help a person to maintain balance of internal human physiological systems (Kawachi & Berkman, 2001; Thoits, 2011). Indirectly, social support can impact health by enhancing the ability to cope with psychosocial stress resulting from adverse life events by lessening situational demands and mitigating emotional reactions to stressors (Thoits, 2011). Therefore, social support can buffer the adverse health effects caused by stress. Social support can also impact health negatively. Stressful responsibilities and social strains can come together with social support or even sometimes relational demands can disguise as social support, which can both contribute to poor health (House et al., 1988; K. D. Pierce & Quiroz, 2019).

### 1.2 Estimating the biological mechanisms of social support affecting health using DNA methylation biomarkers

Although the theory of social support is well understood, its causal biological mechanisms remain less clear. Chronic and cumulative lifetime stress related to lack of social support may affect the hypothalamic-pituitary-adrenal (HPA) axis, resulting in sustained glucocorticoid secretion (Iob et al., 2018). The dysregulation of the HPA axis will suppress immune, reproductive, and digestive function and include a pro-inflammatory phenotype linked to systemic physiological wear and tear. However, some studies examine only a single physiological system at a time. For example, many studies have focused on inflammation biomarkers, such as C-reactive protein and interleukin-6, to establish links between social support and immune function (Kiecolt-Glaser et al., 2010; Uchino et al., 2018; Y. C. Yang et al., 2014). Others have examined biomarkers related to broader stressed-related phenotypes but reported inconsistent findings. For instance, a recent meta-analysis demonstrated no overall effect of social support on telomere length, a potential outcome for stress-related neuroendocrine responses (Kiecolt-Glaser et al., 2010; Montoya & Uchino, 2023). Similarly, a systematic review of research on social support and allostatic load, a measure of the cumulative effects of stress on the body, reported mixed results (Wiley et al., 2017). While these studies provide valuable insights, biomarkers better capturing the multisystemic effects of chronic stress are needed.

Epigenetic changes, particularly in DNA methylation, are sensitive downstream effects of some stress responses. For instance, multiple studies have shown that chronic psychosocial stress modifies DNA methylation patterns at glucocorticoid receptor binding sites and other stress-responsive genomic regions (Leung et al., 2022). DNA methylation–based aging clocks may have the potential to combine stress-related multisystemic effects offering sensitive and scalable indicators of cumulative physiological stress and biological aging. In addition, they measure environmentally responsive methylation changes at DNA sites in multiple genes, and can be trained to predict a wide-range of conditions or age-related outcomes, including broad stress-related outcomes (Horvath & Raj, 2018). These characteristics make DNA methylation clocks promising biomarkers for assessing biological system dysregulation associated with social support. Research is needed to explore the relationship between DNA methylation clocks and social support and whether these epigenetic biomarkers can provide biological signals for long-term disease onset or mortality.

### 1.3 Social support among different groups

The perception or reception of social support varies significantly across individuals due to personal characteristics and social circumstances. Perceived social support has been associated with the perceivers’ personality such as the level of extraversion, neuroticism, self-esteem, and self-verification (G. R. Pierce et al., 1997; Swickert et al., 2010). Studies also suggest that macrosocial cultures give rise to microsocial relationships and supports (House et al., 1988; Taylor & others, 2011). For instance, although there is a great amount of variation within race and ethnicity, studies have shown that on average, compared to Hispanics, African Americans tend to have larger support networks (Mui, 1993), and White Americans tend to have more types of social support available to them (Golding & Burnam, 1990). Compared to White Americans, African Americans tend to have a smaller size of social networks and lower levels of social engagement (Barden et al., 2016; Barnes et al., 2004). The intersection of race and gender also increases the variability of understanding social support (Barnes et al., 2004; Blau et al., 1979; Luca et al., 2024; Szkody et al., 2021). Difference in social support measured between White and African Americans is significantly attenuated within male participants only (Barnes et al., 2004; Blau et al., 1979). Within Mexican Americans, women have fewer social supports relative to men (Blau et al., 1979; Luca et al., 2024). This variability makes it challenging to define and measure the effects of social support accurately. Due to the large variation in social support seen across individual demographics, it is expected that these differences would extend to associated health outcomes as well. To reveal the true effects of social support on health, larger and more representative samples are needed allowing for not only better overall estimates but also estimates relevance for specific demographic subgroups.

Utilizing the data from the U.S. National Health and Nutrition Examination Survey (NHANES), this study provides a unique opportunity to evaluate aging-related hypotheses by integrating multiple social support measures with a diverse set of DNA methylation-based biomarkers in an older, racially and ethnically diverse population. By leveraging NHANES’ large, nationally representative sample and its extensive collection of health-related variables, we can stratify our analyses to assess how these associations differ at the intersections of race, ethnicity, and gender. We hypothesize that DNA methylation-based aging biomarkers will be significantly associated with social support, and these associations will vary across demographic groups. Additionally, NHANES’ longitudinal mortality data allows us to explore whether the epigenetic aging biomarkers mediate the well-established relationship between social support and mortality. These findings will provide preliminary evidence on the potential biological signals through which social support influences longevity.

## 2 Methods

### 2.1 Participants

The US National Health and Nutrition Examination Survey (NHANES) is a large cross-sectional sample survey with a broad range of information collected for each participants, including blood samples, social demographic variables, and psychosocial measures (Tissues, 1991). NHANES collects data from the noninstitutionalized US population in a two-year cycle, and the NHANES 1999–2000 and 2001–2002 cycles were used in this study because DNA methylation data only had been collected in these cycles. Social support variables were only collected among participants who were 60 years and older in this dataset, so anyone who was 60 or older at baseline and had DNA methylation clock measures were included in this study (N = 1838). Participants who were 85 and older were also excluded in this study because the age was top coded in the NHANES dataset for privacy protection (N = 130). Then participants who self-reported as “other Hispanics” or “other race/ethnicity” were excluded for our primary analysis since the sample size of these racial groups did not provide enough statistical power to conduct subgroup analysis by gender (N = 145). Our final sample size was 1,563, including 362 non-Hispanic Blacks (179 women and 183 men), 665 non-Hispanic Whites (324 women and 341 men), and 536 Mexican Americans (254 women and 282 men).

### 2.2 Social support measures

The social support questionnaire in NHANES was adapted from the Yale Health and Aging study (Seeman et al., 2001) and the Berkman-Syme Social Network Index (Berkman & Syme, 1979). The questions, although they do not include every aspect of social support, include many important ones frequently used in previous studies (C. N. Bell et al., 2010; Porch et al., 2016; Shiue, 2015; Smith & Waitzman, 1997; White et al., 2009). These questions include perceived emotional support and perceived financial support representing the functional support. The size of network, which has been shown by studies to be more consequential for health than other quantitative aspects of relationships, captures structural support. Marital relationship status, considered as the most important dyadic social relationship, is also included in the analysis (House et al., 1988). Perceived emotional support was assessed using the question: “Can you count on anyone to provide you with emotional support such as talking over problems or helping you make a difficult decision?”, which was the primary exposure. We used the follow-up question “In the last 12 months, could you have used more emotional support than you received?” to estimate having enough perceived emotional support as a secondary exposure. Anyone answering “no” to this question was categorized as having enough emotional support, with all the other participants considered as not having enough emotional support (Shiue, 2015). Social network size was assessed using the question: “How many close friends (relatives or nonrelatives) do you have?”, with responses ranging from 0 to 50. We reported participants as having any close friends (close friends ≥1) as the primary exposure and having five or more (close friends ≥5) as the secondary exposure. Cutoffs were selected based on prior studies which commonly used (White et al., 2009). Perceived financial support was assessed using the binary question: “If you need some extra help financially, could you count on anyone to help you; for example, by paying any bills, housing costs, hospital visits, or providing you with food or clothes?”. Where participants who answered “yes” were classified as having financial support, while those answering “no” being categorized as no one financial support. Marital status was assessed as a binary variable. Participants who were married or living with a partner were grouped together as “married”, while those who were never married, separated, divorced, or widowed were grouped as “not married” and comprised the reference group for analysis.

### 2.3 Epigenetic aging measures

The Illumina Infinium Methylation EPIC Assay was used to measure the DNA methylation sites in blood samples. Quality control steps included outlier removal, color correction, background subtraction, and normalization. The original beta-mixture quantile (BMIQ) method (Teschendorff et al., 2013) was used to normalize all biomarkers except for Hannum, Horvath, SkinBlood, PhenoAge, GrimAge, and GrimAge2. A modified BMIQ version using a gold standard reference panel produced by Horvath et al. (Horvath et al., 2015) was used for the remaining clocks. DNA methylation clocks were then calculated for each participant using the pre-processed DNA methylation dataset described above. Calculation of the biomarkers are described in further detail on the NHANES website (Centers for Disease Control and Prevention (CDC), 2024). The final 13 DNA methylation-based biomarkers included chronological age clocks (Hannum (Hannum et al., 2013), Horvath (Horvath, 2013), Weidner (Weidner et al., 2014), Lin (Lin et al., 2016), Vidal-Bralo (Vidal-Bralo et al., 2016), SkinBlood (Horvath et al., 2018), and Zhang (Zhang et al., 2019)), physiological functioning biomarkers (Yang (Z. Yang et al., 2016), PhenoAge (Levine et al., 2018), GrimAge (Lu, Quach, et al., 2019), Telomere (Lu, Seeboth, et al., 2019), and GrimAge2 (Lu et al., 2022)), and the pace of aging measure (DunedinPoAm (Belsky et al., 2020)). Though chronological aging clocks were reported to be less associated with social support than other DNA methylation-based biomarkers (Rentscher et al., 2023; Yin et al., 2025), they were still included to assess if results validated among this diverse sample. We also included the DNA methylation-predicted telomere length to confirm the previous findings about telomere length and social support in our sample (Montoya & Uchino, 2023). Consistent results across multiple clocks may suggest strong signals considering the heterogeneity in what these clocks can capture; therefore, we did not exclude any clock available in the NHANES dataset.

### 2.4 Covariates

Social support has been reported to be associated with many factors including demographic, socioeconomic, and health behavioral factors, especially among older adults (Maier et al., 2021; Thoits, 1985). Demographic covariates of age and nativity (US born vs foreign born) were accounted for in our models, including an age^2^ term to account for potential non-linear trends of DNAm with advanced age. Socioeconomic covariates included educational attainment (less than high school, high school/GED, some college or associates degree, and college degree or higher), poverty income ratio (PIR) (<1, 1–2, 2–5, 5+), and the self-reported longest held occupation (white collar and professional, semi-routine white collar, high skill blue collar, semi-routine blue collar, and never worked) (Rehkopf et al., 2008). Previous research suggests that health-related behaviors influenced by social networks may mediate the relationship between social support and health outcomes (Kawachi & Berkman, 2001). However, other studies indicate that certain negative health behaviors, such as smoking and alcohol consumption, can also facilitate social connections (Huang et al., 2014). To illustrate the effects of social support on DNA methylation-based aging biomarkers, independent of behavioral pathways, we adjusted for key health behaviors in our models. The behavioral covariates included smoking status, categorized as nonsmokers, former smokers with less than 30 pack-years, former smokers with 30 or more pack-years, current smokers with less than 30 pack-years, and current smokers with 30 or more pack-years. Alcohol consumption was classified as abstainers versus alcohol drinkers. Physical activity was defined as active versus inactive, with participants considered active if they reported engaging in vigorous, moderate, or muscle-strengthening activities. Diet quality was assessed using the 2015 Healthy Eating Index and categorized into quintiles (quintile 1–5). All data were obtained from publicly available NHANES datasets.

### 2.5 Mortality and blood cell type composition variables

Mortality outcomes were derived from the National Center for Health Statistics public use dataset. Participants were assumed deceased if their death certificate records were linked from the National Death Index, where those without linked death records were assumed alive. Age at date of death or by the end of the follow-up period (until December 31, 2019) was used as the continuous variable for survival outcome. Blood cell-type proportions were obtained from complete blood count differential assays measured directly on the same blood samples used for processing DNA methylation arrays. These proportions were not estimated from the methylation data, but rather reflect actual clinical hematological measurements, including lymphocytes, monocytes, segmented neutrophils, eosinophils, and basophils.

### 2.6 Statistical modeling

Descriptive statistics were reported separately for the intersections of race, ethnicity, and gender. Means and standard deviations were calculated for continuous variables, while counts and percentages were reported for categorical variables. To address missing data in exposure and covariate variables, we implemented a nonparametric random forest multiple imputation approach using the *missForest* package in R. The imputation model was constructed using data from all NHANES 1999–2002 participants (n = 21,004), excluding outcome variables. To examine associations between social support variables and DNA methylation biomarkers, we employed weighted linear regression models using the *survey* package in R. Each model was estimated separately for all combinations of individual social support variables and DNA methylation biomarkers, incorporating strata, primary sampling unit indicators, and NHANES survey weights specifically designed for participants with DNA methylation measures. The main models were adjusted for demographic, socioeconomic, and health behavior covariates. Statistical significance was assessed using a p-value threshold of <0.05. To account for multiple comparisons, a Bonferroni correction was applied, setting the adjusted significant threshold at p < 0.0014 (0.05/36).

For survival analysis, we applied a Weibull regression model using the *survey* package in R. Model-based mediation analyses were conducted using the *mediation* package in R (Tingley et al., 2019), which employs a potential outcome framework to estimate mediation effects. This approach allows for researcher-defined outcome and mediator models while adhering to the following assumptions: 1) No unobserved pre-treatment confounding between social support variables and mortality outcomes, beyond the covariates already controlled for; 2) No unobserved pre-treatment or post-treatment confounding between DNA methylation biomarkers and mortality outcomes; 3) Sufficient common support areas for social support variables and DNA methylation biomarkers, ensuring that after stratification by all covariates, both exposure groups retain adequate observations for sound inference.

To assess the robustness of findings, we conducted additional sensitivity analyses by adjusting for covariates excluding behavioral variables, adding blood cell type composition variables, adding chronic disease covariates including cancer, diabetes, and cardiovascular diseases self-reported clinical diagnosis history, or restricting the original models to complete case analyses. To avoid the potential collinearity issues introduced by including all blood cell type composition variables at the same time, while retaining the full biological information contained in any specific cell type, we applied an isometric log-ratio (ILR) transformation to the original blood cell type composition variables using the *compositions* package in R (Van den Boogaart & Tolosana-Delgado, 2008). ILR transforms the compositional data into an orthogonal coordinate system in Euclidean space, producing D–1 linearly independent variables from D parts. The resulting ILR coordinates were included in the regression models as covariates. This method follows best practices in compositional data analysis and avoids the pitfalls of both raw inclusion and arbitrary omission of components (Gloor et al., 2017). In addition, we did a sensitivity analysis using an overall social support variable called the Social Network Index (SNI) based on the sum of four questions (Berkman & Syme, 1979; Pantell et al., 2013; Rees et al., 2010). These questions included marital status, emotional support, financial support, and whether the participant had 5 or more friends. Each question was scored as either 0 or 1, with 1 indicating the presence of that type of social support, resulting in a total SNI score raging from 0 to 4. We then dichotomized the index according to previous NHANES studies: scores of 0 or 1 were classified as low SNI (most isolated) using as the reference group, and scores of 2, 3, or 4 were classified as high SNI (Fleisch Marcus et al., 2017; Koh-Bell et al., 2021; Luo & Hendryx, 2022; Marcus et al., 2016). We provided additional sensitivity results with combining 0, 1, and 2 into the low SNI group since this analysis should have greater power due to balanced group sample sizes between low SNI and high SNI.

To facilitate comparisons between DNA methylation clocks and previously proposed integrative biomarkers, we calculated both the Chronic Stress Indicator (CSI) and Allostatic Load (AL) following established methods from prior studies (Guidi et al., 2020; Hill et al., 2024), using the raw dataset before imputation. We then examined and reported the correlations between the DNA methylation clocks and both CSI and AL.

Effects sizes were reported as linear regression coefficients, hazard ratios (HRs), and mediated proportions, along with their 95 % confidence intervals (CIs). All analyses were performed using R version 4.4.1, with the R code and data used for these analyses being publicly available in a GitHub repository (https://github.com/shywatson/social_support_DNAm_NHANES).

## 3 Results

### 3.1 Sample statistics

Descriptive statistics are shown in Table 1. Among our participants, the captured death events in each subgroup ranged from 37.0 % to 60.7 %, with the lowest rates among Mexican Americans. More than 90 % of Black and White participants reported having a source of emotional support. For having enough emotional support, White men showed the highest rate (86.5 %) while Mexican American women and men showed the lowest rates (64.6 % and 66.3 %, respectively). A large proportion of participants reported having close friends with Mexican American men reporting the lowest rate (90.1 %). Regarding having five or more close friends, Black participants reported the lowest rates (33.0 % and 32.2 % among women and men, respectively), followed-by Mexican Americans. For having financial support, the rates across subgroups were similar ranging from 70.6 % to 85.5 %. For marital status, Black, White, and Mexican American women had lower rates (33.5 %, 54.9 %, and 53.1 %) compared to men (63.9 %, 81.5 % and 83.0 %, respectively). The missing rates by subgroups and by each covariate ranged from 0.0 % to 22.0 % which are shown in Table S1.

**Table 1:** Descriptive statistics for mortality outcome, social support measures, and demographic, socioeconomic, and behavioral covariates, NHANES 1999–2002 (N = 1563).

Variables used in the study	Black Women (N = 179)		Black Men (N = 183)		White Women (N = 324)		White Men (N = 341)		Mexican American Women (N = 254)		Mexican American Men (N = 282)	
	Count/Mean	Percentage/SD	Count/Mean	Percentage/SD	Count/Mean	Percentage/SD	Count/Mean	Percentage/SD	Count/Mean	Percentage/SD	Count/Mean	Percentage/SD
**Death event captured**	79	(44.1 %)	111	(60.7 %)	163	(50.3 %)	203	(59.5 %)	94	(37.0 %)	130	(46.1 %)

**Social support measures**												

Perception of having any emotional support												
No one emotional support	13	(7.3 %)	17	(9.3 %)	16	(4.9 %)	23	(6.7 %)	32	(12.6 %)	37	(13.1 %)
Having anyone emotional support	166	(92.7 %)	166	(90.7 %)	308	(95.1 %)	318	(93.3 %)	222	(87.4 %)	245	(86.9 %)
Perception of having enough emotional support												
Not enough emotional support	34	(19.0 %)	42	(23.0 %)	65	(20.1 %)	46	(13.5 %)	90	(35.4 %)	95	(33.7 %)
No need more support	145	(81.0 %)	141	(77.0 %)	259	(79.9 %)	295	(86.5 %)	164	(64.6 %)	187	(66.3 %)
Having any close friends												
Do not have close friends	8	(4.5 %)	13	(7.1 %)	9	(2.8 %)	10	(2.9 %)	16	(6.3 %)	28	(9.9 %)
Having 1 or more close friends	171	(95.5 %)	170	(92.9 %)	315	(97.2 %)	331	(97.1 %)	238	(93.7 %)	254	(90.1 %)
Having 5 or more close friends												
Having less than 5 close friends	120	(67.0 %)	124	(67.8 %)	170	(52.5 %)	165	(48.4 %)	161	(63.4 %)	181	(64.2 %)
Having 5 or more close friends	59	(33.0 %)	59	(32.2 %)	154	(47.5 %)	176	(51.6 %)	93	(36.6 %)	101	(35.8 %)
Perception of having any financial support												
No one financial support	26	(14.5 %)	41	(22.4 %)	51	(15.7 %)	88	(25.8 %)	54	(21.3 %)	83	(29.4 %)
Having anyone financial support	153	(85.5 %)	142	(77.6 %)	273	(84.3 %)	253	(74.2 %)	200	(78.7 %)	199	(70.6 %)
Marital status												
Never married, separated, divorced, or widowed	119	(66.5 %)	66	(36.1 %)	146	(45.1 %)	63	(18.5 %)	119	(46.9 %)	48	(17.0 %)
Married or living with a partner	60	(33.5 %)	117	(63.9 %)	178	(54.9 %)	278	(81.5 %)	135	(53.1 %)	234	(83.0 %)

**Demographic covariates**												

Age	68.82	(6.90)	68.01	(5.97)	71.94	(7.27)	71.69	(7.09)	68.04	(6.02)	67.99	(6.11)
Nativity												
US born	169	(94.4 %)	167	(91.3 %)	304	(93.8 %)	325	(95.3 %)	136	(53.5 %)	154	(54.6 %)
Foreign born	10	(5.6 %)	16	(8.7 %)	20	(6.2 %)	16	(4.7 %)	118	(46.5 %)	128	(45.4 %)

**Socioeconomic covariates**												

Educational attainment												
Less than high school	104	(58.1 %)	102	(55.7 %)	77	(23.8 %)	71	(20.8 %)	189	(74.4 %)	225	(79.8 %)
High school/GED	33	(18.4 %)	34	(18.6 %)	122	(37.7 %)	94	(27.6 %)	25	(9.8 %)	17	(6.0 %)
Some college or associates degree	27	(15.1 %)	28	(15.3 %)	81	(25.0 %)	67	(19.6 %)	31	(12.2 %)	28	(9.9 %)
College degree or higher	15	(8.4 %)	19	(10.4 %)	44	(13.6 %)	109	(32.0 %)	9	(3.5 %)	12	(4.3 %)
Family income												
PIR<1	35	(19.6 %)	31	(16.9 %)	27	(8.3 %)	18	(5.3 %)	62	(24.4 %)	64	(22.7 %)
PIR 1-2	63	(35.2 %)	60	(32.8 %)	87	(26.9 %)	62	(18.2 %)	105	(41.3 %)	120	(42.6 %)
PIR 2-5	67	(37.4 %)	76	(41.5 %)	152	(46.9 %)	175	(51.3 %)	68	(26.8 %)	80	(28.4 %)
PIR 5+	14	(7.8 %)	16	(8.7 %)	58	(17.9 %)	86	(25.2 %)	19	(7.5 %)	18	(6.4 %)
Longest held occupation												
White collar and professional	31	(17.3 %)	23	(12.6 %)	91	(28.1 %)	133	(39.0 %)	26	(10.2 %)	31	(11.0 %)
White collar, semi-routine	38	(21.2 %)	21	(11.5 %)	127	(39.2 %)	46	(13.5 %)	34	(13.4 %)	13	(4.6 %)
Blue collar, high skill	15	(8.4 %)	38	(20.8 %)	19	(5.9 %)	77	(22.6 %)	40	(15.7 %)	63	(22.3 %)
Blue collar, semi-routine	89	(49.7 %)	93	(50.8 %)	76	(23.5 %)	78	(22.9 %)	147	(57.9 %)	163	(57.8 %)
Never worked	6	(3.4 %)	8	(4.4 %)	11	(3.4 %)	7	(2.1 %)	7	(2.8 %)	12	(4.3 %)

**Behavioral covariates**												

Smoking												
Never smoker	118	(65.9 %)	58	(31.7 %)	181	(55.9 %)	108	(31.7 %)	166	(65.4 %)	87	(30.9 %)
Former smoker less than 30 pack years	35	(19.6 %)	47	(25.7 %)	51	(15.7 %)	97	(28.4 %)	54	(21.3 %)	98	(34.8 %)
Former smoker 30+ pack years	10	(5.6 %)	34	(18.6 %)	62	(19.1 %)	98	(28.7 %)	15	(5.9 %)	47	(16.7 %)
Current smoker less than 30 pack years	8	(4.5 %)	15	(8.2 %)	4	(1.2 %)	11	(3.2 %)	16	(6.3 %)	24	(8.5 %)
Current smoker 30+ pack years	8	(4.5 %)	29	(15.8 %)	26	(8.0 %)	27	(7.9 %)	3	(1.2 %)	26	(9.2 %)
Alcohol consumption												
Abstainer	117	(65.4 %)	82	(44.8 %)	145	(44.8 %)	117	(34.3 %)	147	(57.9 %)	111	(39.4 %)
Alcohol drinker	62	(34.6 %)	101	(55.2 %)	179	(55.2 %)	224	(65.7 %)	107	(42.1 %)	171	(60.6 %)
Physical activity												
Inactive	121	(67.6 %)	103	(56.3 %)	158	(48.8 %)	121	(35.5 %)	166	(65.4 %)	154	(54.6 %)
Active	58	(32.4 %)	80	(43.7 %)	166	(51.2 %)	220	(64.5 %)	88	(34.6 %)	128	(45.4 %)
2015 healthy eating index												
1st quintile	62	(34.6 %)	82	(44.8 %)	107	(33.0 %)	106	(31.1 %)	70	(27.6 %)	82	(29.1 %)
2nd quintile	9	(5.0 %)	12	(6.6 %)	16	(4.9 %)	23	(6.7 %)	13	(5.1 %)	17	(6.0 %)
3rd quintile	11	(6.1 %)	9	(4.9 %)	22	(6.8 %)	20	(5.9 %)	7	(2.8 %)	11	(3.9 %)
4th quintile	20	(11.2 %)	16	(8.7 %)	25	(7.7 %)	34	(10.0 %)	22	(8.7 %)	28	(9.9 %)
5th quintile	77	(43.0 %)	64	(35.0 %)	154	(47.5 %)	158	(46.3 %)	142	(55.9 %)	144	(51.1 %)

### 3.2 Perception of having any emotional support and enough emotional support

**Fig. 1:**
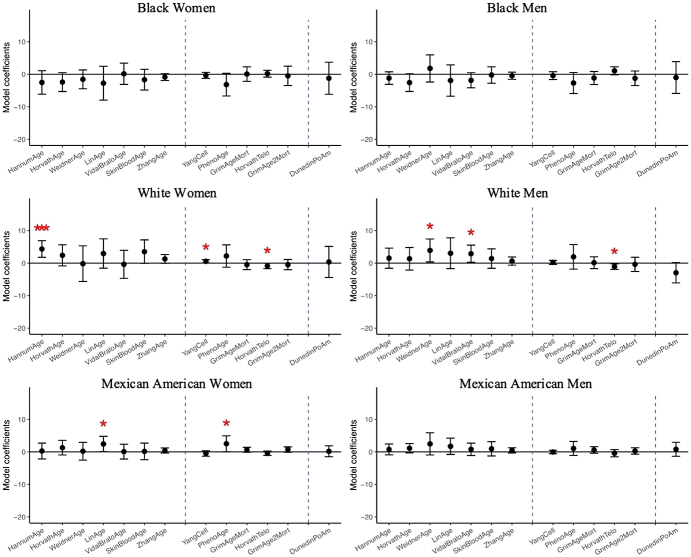
Linear regression coefficients for perception of having any emotional support on DNA methylation clocks controlling for demographic, socioeconomic, and behavioral covariates, N = 1563. Coefficients with one asterisk indicates statistical significance (p-value<0.05), and three asterisks indicated significance passed Bonferroni adjustment (p-value<0.0014). To visualize coefficients which differ in scale in a way that makes them comparable to each other, the coefficients of Yang and DunedinPoAm were multiplied by 100 and Telomere length by 10.

The associations between the perception of having a source of emotional support and epigenetic aging biomarkers, adjusted for demographic, socioeconomic, and behavioral covariates, are presented in Fig. 1 and Table S2. Among White women, having emotional support was significantly associated with higher Hannum (*b* = 4.34 years; 95 %CI: 1.78,6.90) and Yang (*b* = 0.005; 95 %CI: 0.000,0.010) epigenetic age, as well as shorter DNA methylation-predicted telomere length (*b* = −0.09 kilobase pairs (kbp); 95 %CI: −0.17,-0.00). Similarly, among White men, having emotional support was significantly associated with higher Weidner (*b* = 3.89 years; 95 %CI: 0.36,7.42) and Vidal-Bralo (*b* = 2.89 years; 95 %CI: 0.25,5.53) epigenetic age, along with shorter predicted telomere length (*b* = −0.11 kbp; 95 %CI: −0.19,-0.03). Among Mexican American women, having a source of emotional support was significantly associated with higher Lin (*b* = 2.44 years; 95 %CI: 0.10,4.77) and PhenoAge (*b* = 2.52 years; 95 %CI: 0.06,4.97) epigenetic age. No significant associations were observed between perceived emotional support and any DNA methylation aging biomarkers among Mexican American men, Black men, or Black women.

The associations between the perception of having enough emotional support and DNA methylation aging biomarkers are presented in Fig. S1 and Table S3. Apart from a significant finding among White men with higher Vidal-Bralo epigenetic age (*b* = 2.25 years; 95 %CI: 0.22,4.28), significant associations were seen only among Black men. In this group, there was a consistent trend of associations between perception of enough emotional support and slower epigenetic aging with the largest effect reflected on PhenoAge epigenetic age (*b* = −3.78 years; 95 %CI: −6.35,-1.21).

### 3.3 Having any close friends and having five or more close friends

**Fig. 2:**
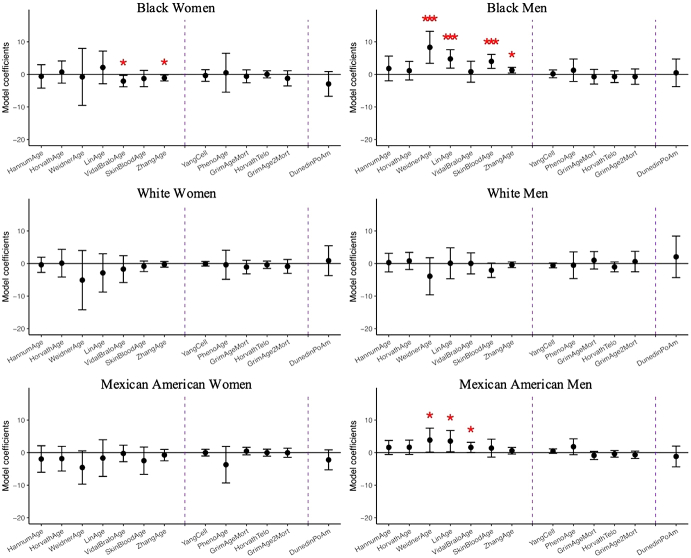
Linear regression coefficients for having any close friends on DNA methylation clocks controlling for demographic, socioeconomic, and behavioral covariates, N = 1563. Coefficients with one asterisk indicates statistical significance (p-value<0.05), and three asterisks indicated significance passed Bonferroni adjustment (p-value<0.0014). To visualize coefficients which differ in scale in a way that makes them comparable to each other, the coefficients of Yang and DunedinPoAm were multiplied by 100 and Telomere length by 10.

The associations between having any close friends and DNA methylation aging biomarkers are shown in Fig. 2 and Table S4. Among men from minoritized groups, having any close friends (compared to having none) was associated with faster epigenetic aging, particularly among the chronological age-based DNA methylation clocks. Among Black men, having any close friends was significantly associated with higher Weidner (*b* = 8.30 years; 95 %CI: 3.39,13.21), Lin (*b* = 4.75 years; 95 %CI: 1.93,7.56), Skinblood (*b* = 3.98 years; 95 %CI: 1.84,6.13), and Zhang (*b* = 1.29 years; 95 %CI: 0.43,2.15) epigenetic age. Similarly, among Mexican American men, having any close friends was associated with higher Weidner (*b* = 3.86 years; 95 %CI: 0.18, 7.53), Lin (*b* = 3.54 years; 95 %CI: 0.28, 6.80), and Vidal-Bralo epigenetic age (*b* = 1.60 years; 95 %CI: 0.05, 3.16). In contrast, among Black women, having any close friends was associated with slower epigenetic aging when observing Vidal-Bralo (*b* = −2.07 years; 95 %CI: −3.83,-0.32) and Zhang (*b* = −1.10 years; 95 %CI: −2.00,-0.20) epigenetic age.

Regarding having five or more close friends, the associations with DNA methylation aging biomarkers are shown in Fig. S2 and Table S5. Compared to the findings for any close friends, the associations trended toward null for Black participants and Mexican American men, with the exception that GrimAge and GrimAge2 became significant associated among Mexican American men. However, among White women, having five or more close friends was significantly associated with slower epigenetic aging, as indicated by decreased Weidner (*b* = −3.21 years; 95 %CI: −5.17,-1.24) and Yang (*b* = −0.004; 95 %CI: −0.007,-0.000) epigenetic age. Conversely, among Mexican American women, having five or more close friends was marginally associated with faster epigenetic aging on higher Hannum (*b* = 1.27 years; 95 %CI: 0.01,2.53) and SkinBlood (*b* = 1.30 years; 95 %CI: 0.11,2.48) epigenetic age.

### 3.4 Perception of having any financial support and marital status

**Fig. 3:**
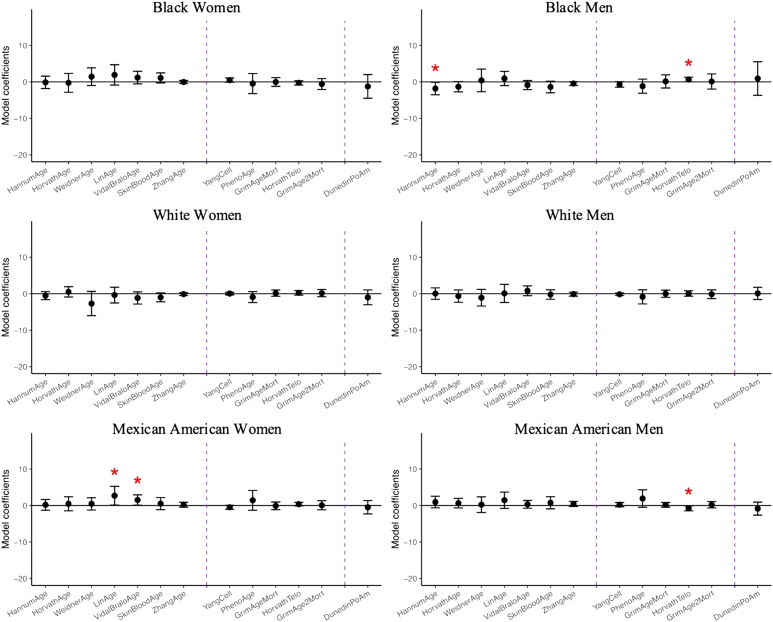
Linear regression coefficients for perception of having any financial support on DNA methylation clocks controlling for demographic, socioeconomic, and behavioral covariates, N = 1563. Coefficients with one asterisk indicates statistical significance (p-value<0.05), and three asterisks indicated significance passed Bonferroni adjustment (p-value<0.0014). To visualize coefficients which differ in scale in a way that makes them comparable to each other, the coefficients of Yang and DunedinPoAm were multiplied by 100 and Telomere length by 10.

As shown in Fig. 3 and Table S6, the perception of having a source of financial support exhibited weaker associations with DNA methylation-based aging biomarkers compared to having emotional support and close friends. Among Black men, having financial support was significantly associated with slower epigenetic aging on Hannum epigenetic age (*b* = −1.83 years; 95 %CI: −3.53,-0.13) and predicted telomere length (*b* = 0.06 kbp; 95 %CI: 0.00,0.13). In contrast, for Mexican Americans, having financial support was associated with faster epigenetic aging. Among Mexican American women, Lin and Vidal-Bralo displayed increased effects with *b* = 2.70 years (97 %CI: 0.12,5.27) and *b* = 1.52 years (95 %CI: 0.10,2.95), respectively. Among Mexican American men, having financial support was associated with shorter predicted telomere length (*b* = −0.08 kbp; 95 %CI: −0.14,-0.01).

**Fig. 4:**
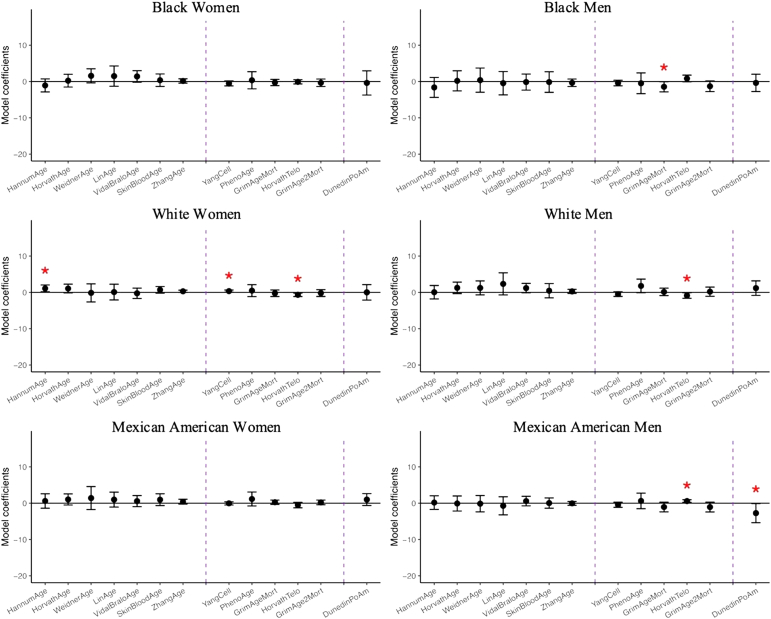
Linear regression coefficients for marital status on DNA methylation clocks controlling for demographic, socioeconomic, and behavioral covariates, N = 1563. Coefficients with one asterisk indicates statistical significance (p-value<0.05), and three asterisks indicated significance passed Bonferroni adjustment (p-value<0.0014). To visualize coefficients which differ in scale in a way that makes them comparable to each other, the coefficients of Yang and DunedinPoAm were multiplied by 100 and Telomere length by 10.

The associations between DNA methylation aging biomarkers and marital status are shown in Fig. 4 and Table S7. Being married or living with a partner was associated with lower epigenetic age across men from minoritized groups. Among Black men, being married was significantly associated with lower GrimAge epigenetic age (*b* = −1.45 years; 95 %CI: −2.83,-0.06), and among Mexican American men, it was associated with longer predicted telomere length (*b* = 0.06 kbp; 95 %CI: 0.02,0.10) and lower DunedinPoAm measure (*b* = −0.027; 95 %CI: −0.054,-0.001). Contrarily, among White participants, being married or living with a partner was associated with increased epigenetic aging. In both White men and women, being married was linked to shorter predicted telomere length (*b* = −0.09 kbp; 95 %CI: −0.16,-0.01 and *b* = −0.07 kbp; 95 %CI: −0.12,-0.02, respectively). Additionally, among White women, being married was associated with higher Hannum (*b* = 1.09 years; 95 %CI: 0.13,2.05) and Yang (*b* = 0.003; 95 %CI: 0.000,0.007) epigenetic age.

### 3.5 Mediation analysis

In mediation analyses, Hannum epigenetic age and DNA methylation-predicted telomere length significantly and negatively mediated the relationship between perceived emotional support and mortality among White women, with mediated proportions of −20.9 % (95 %CI: −51.0 %, −7.0 %) and −9.1 % (95 %CI: −27.6 %,-0.6 %), respectively (results not shown in tables). The negative mediation effects suggest suppressor effects, where causal mediation and direct effects operate in opposite directions. While perceived emotional support was overall protective against mortality among White women, its pathway through increased Hannum epigenetic age or shortened predicted telomere length appeared harmful. The total effect of perceived emotional support on mortality in White women after adjusting for demographic, socioeconomic, and behavioral covariates was HR = 0.29 (95 %CI: 0.16,0.53) (Table S8). After adjusting for either Hannum epigenetic age or predicted telomere length, the direct effects strengthened to 0.23 (95 %CI: 0.12,0.45) and 0.27 (95 %CI: 0.15, 0.50), respectively (results not shown in tables). No other DNA methylation aging biomarker demonstrated significant mediation effects.

To better understand these mediation effects, the results of the associations between mediators and mortality outcome are shown in Table S9. Due to sample size limitations, none of the DNA methylation aging biomarkers showed statistically significant harmful effects on mortality across all subgroups. Additionally, physiological functioning and the pace-of-aging biomarkers showed more stable associations across subgroups compared to chronological age clocks, although no clock showed any significant associations with mortality among Mexican American men. All our clocks were significantly associated with morality in our combined sample, except Zhang and Yang epigenetic age. For additional information on these clocks, Table S10 presents more details including their training purposes, demographics of their training sets, the tissue(s) examined, and number of probes included.

### 3.6 Sensitivity analysis

The sensitivity results from models only adjusting for demographic and socioeconomic covariates (and not behavioral variables presumed to potentially be on the pathway between social support and DNA methylation) are included in Table S11-S16. The associations between social support variables and DNA methylation clocks were primarily more protective, especially for GrimAge, GrimAge2, and DunedinPoAm. For example, among White men, although having emotional support remained positively associated with the chronological clocks, it showed protective effects on GrimAge, GrimAge2, and DunedinPoAm epigenetic age with −2.02 years (95 %CI: −3.52,-0.52), −2.62 years (95 %CI: −4.23,-1.02), and −0.066 (95 %CI: −0.101,-0.031) respectively (Table S11). Regarding to the mediation analysis results, Hannum and DNA methylation-predicted telomere length showed significant negative mediation effects of emotional support on mortality among White women with a mediated proportion of −18.5 % (95 %CI: −49.0 %, −5.4 %) and −9.1 % (95 %CI: −27.7 %, −0.8 %), respectively. In addition, PhenoAge demonstrated a significant positive mediation effect (mediated proportion: 33.3 %; 95 % CI: 8.4 %, 129.6 %) on the association between having enough emotional support and mortality among Black men. Similarly, GrimAge2 showed a significant positive mediation effect (mediated proportion: 31.0 %; 95 % CI: 1.5 %, 187.9 %) on the association between being married and mortality in Black men too. These positive mediation effects indicate that parts of the total protective effects without controlling for behavioral variables of having enough emotional support (HR = 0.70, 95 %CI: 0.47,1.03) and being married (HR = 0.74, 95 %CI: 0.53,1.02) on Black men are through the pathway of lower PhenoAge or GrimAge2 epigenetic ages. After controlling for each clock, the direct effects reduced to HR = 0.74 (95 %CI: 0.53,1.04) and HR = 0.78 (95 %CI: 0.56,1.09), respectively.

The sensitivity results showed similar patterns after adjusting for additional blood cell type isometric log-ratio (ILR) transformation variables (Table S17-S22). After adjusting for blood cell composition ILR variables, the mediation effects of Hannum epigenetic age and predicted telomere length on perception of emotional support and mortality among White women weakened, with only Hannum epigenetic age retaining statistical significance (mediated proportion: −19.4 %; 95 %CI: −55.6 %,-6.1 %). The sensitivity results from models adjusting for additional chronic disease covariates (non-imputed) were included in Table S23-S28. Most associations between social support variables and DNA methylation clocks remained statistically significant even after adjusting for these chronic disease histories. For the mediation results, the effects of Hannum epigenetic age on perception of emotional support and mortality among White women stayed significant (mediated proportion: −22.7 %; 95 %CI: −57.7 %,-6.9 %).

When restricting the analyses to complete cases (N ranged 1077 - 1117; Table S29-S34), although some associations between social support measures and DNA methylation epigenetic biomarkers mostly got stronger and some got weaker, the bidirectional associations across different racial, ethnic, and gender backgrounds were the same. For example, the effects of perceived financial support on epigenetic clocks among Black men and Mexican American women moved to null, there are protective effects on White men with harmful effects on Mexican American men (Table S33). For the mediation analysis using complete cases, only Hannum epigenetic age remained significant, with the mediated proportion of −19.7 % (95 %CI: −49.5 %,-6.0 %) in the relationship between perceived emotional support and mortality among White women. However, an additional significant mediation effect emerged for GrimAge2 in the association between having close friends and mortality among Black women, with a mediated proportion of 32.0 % (95 %CI: 5.6 %,157.6 %). The total effect after adjusting for demographic, socioeconomic, and behavioral covariates of having close friends on mortality among Black women was HR = 0.32 (95 %CI: 0.11,0.91) using the complete-case sample, while the direct effect was HR = 0.42 (95 %CI: 0.14,1.29).

The results from Social Network Index (SNI) were presented in Table S35. As shown in the table high SNI was associated with lower epigenetic aging among Black men but associated with faster epigenetic aging among White men and Mexican Americans. In addition, sensitivity analysis applying an SNI threshold of 2 yielded results showing additional protective effects among Black women and White participants (Table S36). The harmful effect among Mexican American women remains, yet the harmful effect among Mexican American men was gone. These SNI results should be interpreted with caution and future studies are needed to validate the results since they are sensitive to the threshold used. Models among full sample (including participants self-reported as other Hispanic or race), by each gender, and by each race were presented in Table S37-S73. Although most coefficients demonstrated unclear findings due to heterogeneity of associations across population subgroups, we want to provide these results for the interest of our audiences and for inclusion in future meta-analysis studies. Table S74 showed the correlation results between DNA methylation clocks and both continuous and binary Chronic Stress Indicator (CSI) and Allostatic Load (AL). Compared to AL, CSI showed stronger associations with DNA methylation clocks, especially with Yang and DunedinPoAM measures (r = 0.11 and r = 0.17, respectively).

## 4 Discussion

This study provides preliminary evidence on the associations between DNA methylation aging biomarkers and social support across different racial, ethnic, and gender groups. Our results highlight the complex and heterogeneous nature of these associations, with differential effects observed across subgroups and social support types. Among the various social support measures, having any close friends exhibited the strongest associations with epigenetic aging across all participants. Interestingly, chronological age predictive DNA methylation clocks, particularly Hannum and Weidner, were the most sensitive to social support variability, although these clocks tended to be more weakly associated with disease-related risk factors compared to other types of clocks in previous work (Li et al., 2020). Black men and White women displayed the most numerous associations between several social support measures and DNA methylation clocks. Additionally, our mediation analyses suggested that Hannum epigenetic age, DNA methylation predicted-telomere length, and GrimAge2 epigenetic age may partially mediate the associations between social support and long-term mortality among White or Black women.

Our findings indicate that social support may influence DNA methylation epigenetic aging differently for people based on racial, ethnicity, and gender backgrounds. This finding is important for understanding both the biological signals involved in how social support impacts health and to caution against generalized and over-simplified explanations for how social support may matter for health. For example, according to our results, Black men seemed to benefit more from having enough emotional support than from having five or more close friends, and conversely White women benefited more from having five or more close friends than from having a source of enough emotional support. Previous evidence suggests that functional support such as availability of emotional support may primarily operate through the stress-buffering pathway, while structural support such as social network may mainly operate through the direct pathway (Kawachi & Berkman, 2001). Therefore, our results indicate that Black men may potentially benefit more from the stress-buffering pathway of social support, and White women may benefit more from the direct pathway. This seems to be consistent with results shown from previous studies. Qualitative studies among Black men have shown expressiveness can be reframed as something authentically masculine and cool (Jackson, 2012; Majors & Billson, 1993). In the face of alienation and discrimination that Black men are exposed to, prior work suggests that social support can promote resilience to these exposures in ways that are health promoting (Nguyen et al., 2018). Studies also show presence of a peer companion may not help White women with handling stressful tasks which indicates that White women may benefit less from the stress-buffing pathway (Allen et al., 1991; Snapp, 1992).

Several studies have demonstrated that the meaning and impact of social support vary across demographic groups, further supporting the context-dependent effects of social support. For example, White men may be more negatively impacted by interpersonal conflicts than benefit from positive social support (Lincoln et al., 2003), which could explain the weaker protective effects among those who did not have any emotional support. As social support among White women, especially those working class White women, typically comes from outside of the family (Snapp, 1992). This may be the reason why in our study relationship observed between marriage and higher epigenetic aging among this subgroup. The harmful effects of emotional support among White women, especially the suppressor pattern observed with the Hannum clock, suggest that emotional support can also be a source of strain. This may not only be explained by interpersonal social conflicts, but also can be explained by emotional labor caused by managing emotions to meet social expectations without changing inner feelings which is reported to be more prevalent in women (Wharton, 2009). In contrast to White women, Black women exhibited slower epigenetic aging when they had any close friends in our study. This suggests Black women also benefits from the direct pathway with a smaller social network which is consistent with a previous study (Silverstein & Waite, 1993). For Mexican Americans, prior research suggests that emotional problems are more commonly discussed with a family member rather than a friend (Seeman et al., 2001). Less than 5 % of Mexican Americans consider “compadres” (close friends) as important sources of emotional support (Keefe et al., 1979). This may explain the reason why marriage is the only social support measure showing protective effects on DNA methylation biomarkers in our study among Mercian Americans.

Taken together, these findings align with theories of cultural norms and gendered social roles that shape how support is given and received (Kim et al., 2008; Umberson et al., 1992). For example, one explanation may be that individualistic cultures may emphasize explicit emotional support, such as discussing personal challenges with friends, particularly among women, whereas collectivistic cultures may rely on implicit support from close family relationship, such as marriage, especially for men (Kim et al., 2008). This framework may help explain the beneficial effects of friendship among White and Black women and the protective effects of marriage among Mexican American men observed in our study. Moreover, consistent with prior work, financial/material support affects health outcomes less compared to emotional support (Loprinzi & Joyner, 2016; Thoits, 1985). Overall, our findings highlight both beneficial and detrimental health consequences social support may bring. While social support has been widely recognized as beneficial, prior studies have often reported null results which may be explained by this bidirectional effect on health. Our results emphasize the need for future studies to identify both positive and negative effects of social support and consider the underlying biological mechanisms through which it may influence health.

It is interesting to notice that in our combined sample, Zhang and Yang epigenetic biomarkers did not show significant associations with long-term mortality. Previous studies suggested that DNA methylation biomarkers are important tools for estimating biological aging; outcomes such as prognosis of diseases, recovery and drug response are important indicators of aging too besides long-term survival outcomes (Moqri et al., 2023). For example, Yang clock is specifically designed to estimate stem cell aging related to cancer development; therefore, may not correlate with long-term mortality among healthy sample (Z. Yang et al., 2016). Our sensitivity analyses also revealed mixed directions of association across clocks, particularly when behavioral factors were not controlled for. Behavioral pathways such as smoking and alcohol consumption are well-established mediators linking social support and health outcomes and are closely tied to several DNA methylation clocks but not others (Belsky et al., 2020; Lu et al., 2022). For example, among White men, emotional support was associated with lower GrimAge, GrimAge2, and DunedinPoAm epigenetic age with higher Weidner, Vidal-Bralo, and DNA predicted Telomere length values (Table S11). However, the protective associations disappeared once behavioral covariates were included (Table S2). This pattern suggests that emotional support may promote healthier behaviors among White men, while simultaneously influencing other physiological or psychosocial pathways that counteract these benefits.

The most consistent findings across all subgroups observed in our study were the associations between social support and chronological age predictive DNA methylation clocks Hannum, Weidner, Lin, and Vidal-Bralo, something not previously found (Rentscher et al., 2023; Yin et al., 2025). Overlap in the training datasets of Weidner, Lin, and VidalBralo may explain the clustering of these three clocks. It is noteworthy that these clocks use relatively few DNA methylation sites, suggesting that increasing the amount of features in future epigenetic predictors does not necessarily improve their sensitivity to specific exposures (C. G. Bell et al., 2019; Zhang et al., 2019). Interestingly, Hannum and DNA methylation-predicted telomere length, trained in a mixed sample of White and Hispanic participants and White and Black participants, respectively (Needham et al., 2025), exhibited strong associations across White, Black, and Hispanic groups. This suggests that these clocks can retain predictive performance among participants from ancestry groups that they were not originally trained on. Future research should aim to replicate these findings in independent cohorts and assess their robustness across diverse populations.

While DNA methylation predictors contain sites selected to best predict a given outcome rather than infer causal relationships, it is likely that these sites are involved in the biological aging process. For example, the Weidner clock is based on three DNA sites within genes that regulate pluripotent stem cell function (Liu et al., 2022), suggesting that higher Weidner epigenetic age may reflect replicative exhaustion in the hematopoietic stem cells (Weidner et al., 2014). Additionally, animal studies suggest that social stress can activate extramedullary hematopoiesis, leading to increased neutrophil, erythroid, and myeloid lineage blood cell production (McKim et al., 2018). These findings raise the possibility that social support may influence health through hematopoietic and immune system pathways, a hypothesis that warrants further investigation.

Despite its contributions, this study is not without limitations. First, the social support and DNA methylation measures were collected around the same time making NHANES cross-sectional. This precludes us from determining the directionality, and therefore causality, of any relationships reported. For example, worse health may lead to the marshalling of social resources such as more social support (Gottlieb, 1987) which may be one reason why we see these unexpected positive correlations. Another example is that the relationship observed may be confounded by earlier life experiences which can be associated to both increased social support exposures and faster biological aging. Second, the relatively small subgroup sample sizes may have limited the statistical power, preventing the detection of additional significant associations. Many of our results did not survive multiple testing corrections, which also suggests our results may induce false positives and can only provide exploratory evidence. This is particularly relevant for our subgroup mediation analyses, where wide confidence intervals indicate substantial uncertainty around the estimated effect sizes. Overall, this current study lays the groundwork for the implementation of future studies to replicate and validate these results in larger and more diverse cohorts. Third, our study provides results among people aged 60 years and older due to the availability of NHANES social support variables. This may introduce potential survivor bias since people with low social support among this age group may need to have more unmeasured healthy lifestyle exposures to be selected in the study. This may be another reason why we observed the positive associations between social support and DNA methylation aging biomarkers. Further studies are needed to validate our results among younger populations. Fourth, despite controlling for key socioeconomic and behavioral confounders, residual confounding remains a possibility. Moreover, NHANES did not have measures on negative social interactions and social strains, which can be moderately correlated with social support (Rentscher et al., 2022). This prevents us from differentiating whether negative social experiences or residual confounding causing harmful epigenetic effects detected from this study. Future studies should adopt richer study designs to disentangle these relationships. Fifth, while new DNA methylation-based epigenetic age estimation methods have been developed in recent years, these data are not yet available in the NHANES dataset. For example, principal component–based versions of DNA methylation clocks have been reported to reduce technical noise and improve reliability (Higgins-Chen et al., 2022). These biomarkers were not calibrated for social exposures and lack mechanistic specificity. Therefore, our findings should not be interpreted as mechanistic but rather as aging biomarkers of social support. Future studies should investigate these newer epigenetic clocks to further explore and replicate the associations with social support measures as well as gene specific signatures. Lastly, the NHANES social support variables were collected between 1999 and 2002 and may not fully reflect contemporary social realities. Over the past two decades, particularly following the COVID pandemic, profound changes in economic structures, communication technologies, and cultural norms have likely reshaped how social support is formed, maintained, and experienced (Verdery & Campbell, 2019). Future research should examine how these evolving social conditions influence both the availability and the effects of social support, and whether the forms of structural and functional support that promote well-being or generate strain have shifted over time.

## 5 Conclusions

This study extends the literature by examining the relationships between DNA methylation-based aging biomarkers and social support across racial, ethnic, and gender intersections. Surprisingly, our results suggest social support is predominantly associated with faster epigenetic aging. Specifically, having any emotional support is associated with increased epigenetic age among Mexican American women and White participants, while having close friends is associated with faster epigenetic aging among Black and Mexican American men. Future studies should replicate these findings in larger cohorts with longitudinal study designs. With further validation, DNA methylation-based epigenetic aging biomarkers may serve as short-term surrogate outcomes in future social support research, offering novel insights into the biological mechanisms linking social supports and health.

## CRediT authorship contribution statement

**Hanyang Shen:** Writing – original draft, Visualization, Validation, Methodology, Formal analysis, Data curation, Conceptualization. **Nicole Gladish:** Writing – review & editing, Visualization, Methodology, Formal analysis, Data curation, Conceptualization. **Andres Cardenas:** Writing – review & editing, Methodology. **Belinda L. Needham:** Writing – review & editing, Supervision, Project administration, Methodology, Funding acquisition, Conceptualization. **David H. Rehkopf:** Writing – review & editing, Supervision, Project administration, Methodology, Investigation, Funding acquisition, Conceptualization.

## Ethical Statement

This study used all publicly available data so ethics approval not required.

## Declaration of competing interest

The authors declare that they have no known competing financial interests or personal relationships that could have appeared to influence the work reported in this paper.

## Data Availability

All data used is publicly available.
