# Supplementary material for: Social support and epigenetic aging at the intersections of race, ethnicity, and gender: findings from NHANES 1999–2002
Source: SSM Popul Health. 2025 Dec 4;33:101892. doi: 10.1016/j.ssmph.2025.101892 (PMC12743543; doi:10.1016/j.ssmph.2025.101892)

**Figure S1.** Linear regression coefficients for perception of having enough emotional support on DNA methylation clocks controlling for demographic, socioeconomic, and behavioral covariates, N=1,563. Coefficients with one asterisk indicates statistical significance (p-value<0.05), and three asterisks indicated significance passed Bonferroni adjustment (p-value<0.0014). To visualize coefficients which differ in scale in a way that makes them comparable to each other, the coefficients of Yang and DunedinPoAm were multiplied by 100 and Telomere length by 10.


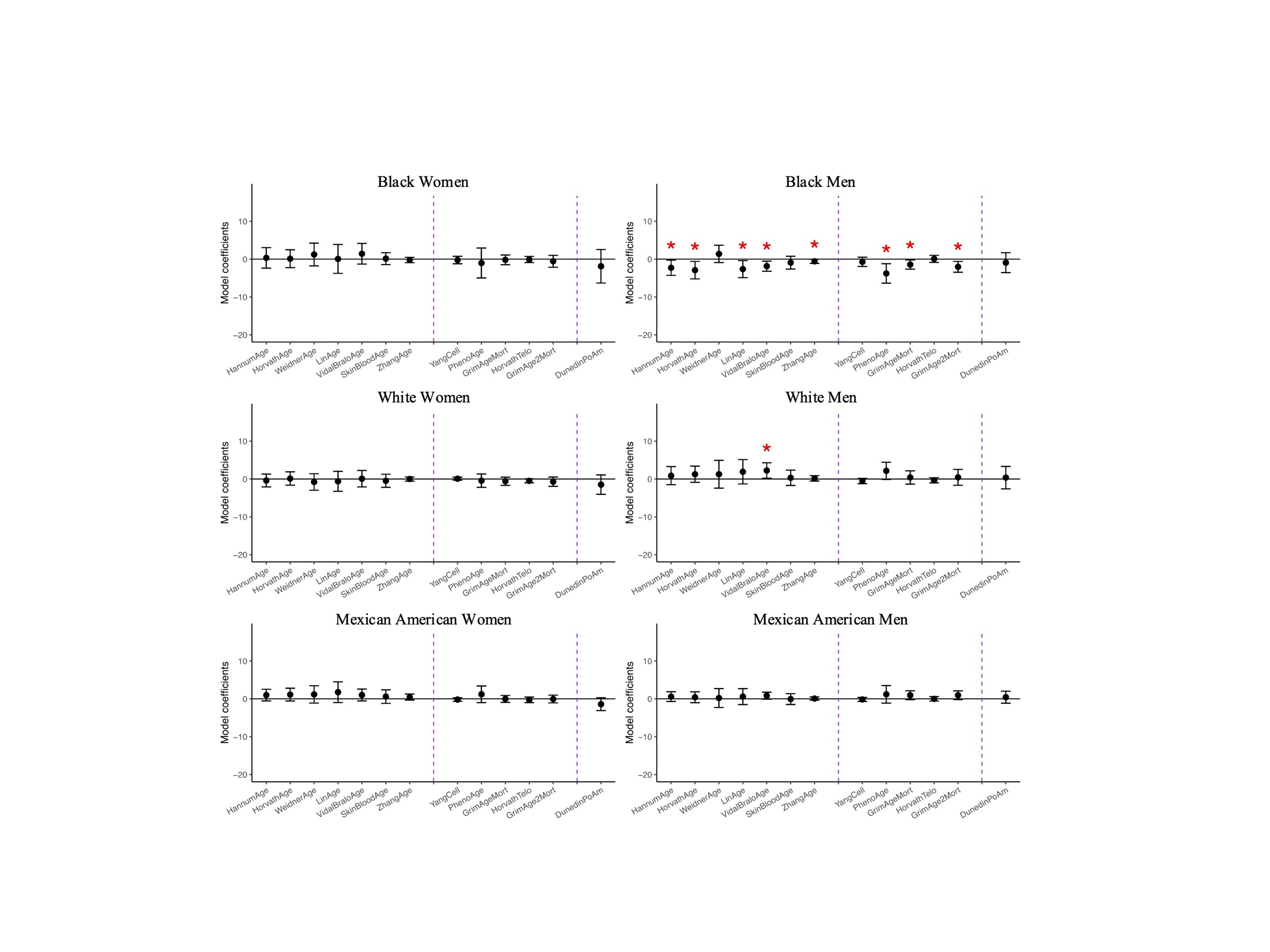


**Figure S2.** Linear regression coefficients for having 5 or more close friends on DNA methylation clocks controlling for demographic, socioeconomic, and behavioral covariates, N=1,563. Coefficients with one asterisk indicates statistical significance (p-value<0.05), and three asterisks indicated significance passed Bonferroni adjustment (p-value<0.0014). To visualize coefficients which differ in scale in a way that makes them comparable to each other, the coefficients of Yang and DunedinPoAm were multiplied by 100 and Telomere length by 10.


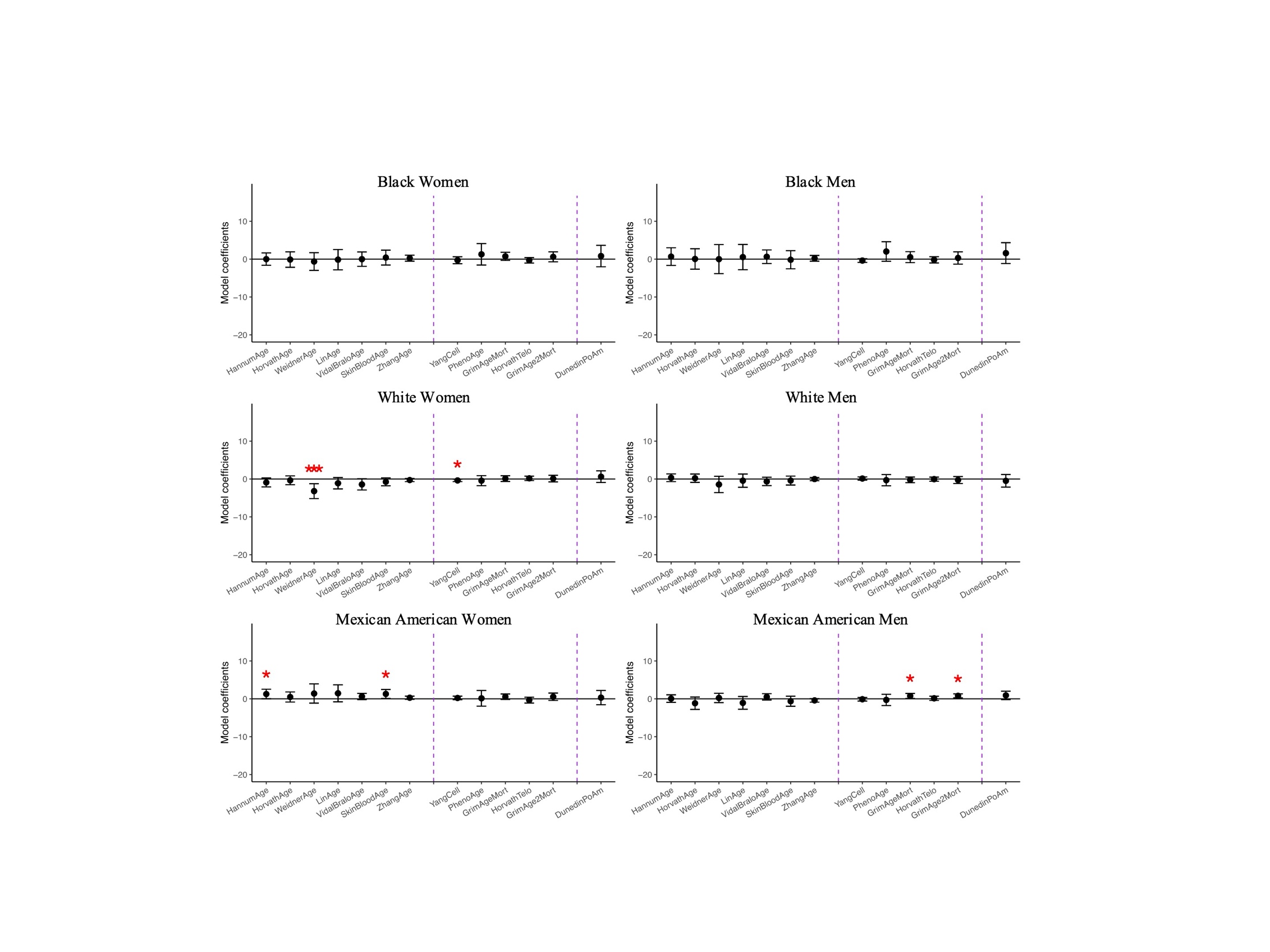

Supplement: Multimedia component 1 [file mmc1.docx]
